# Ascites Presenting as a Peritonitis‐Like Manifestation and Liver Injury due to Trabectedin Monotherapy in a Patient With Myxoid Liposarcoma

**DOI:** 10.1002/cnr2.70740

**Published:** 2026-10-31

**Authors:** Tatsuyuki Kawahara, Hiromichi Yamane, Ayaka Mimura, Hirohito Kirishi, Yusuke Sunada, Yoko Kosaka, Naruhiko Ichiyama, Yasunari Nagasaki, Nobuaki Ochi, Hidekazu Nakanishi, Hideyo Fujiwara, Nagio Takigawa

**Affiliations:** ^1^ Department of General Internal Medicine 4 Kawasaki Medical School Okayama Japan; ^2^ Department of Pathology Kawasaki Medical School Okayama Japan

**Keywords:** ascites presenting as a peritonitis‐like manifestation, liver injury, myxoid liposarcoma, trabectedin

## Abstract

**Background:**

Trabectedin is a synthetic, marine‐derived antitumor agent that exerts cytotoxic effects by binding to the minor groove of DNA. While trabectedin has demonstrated efficacy in advanced soft tissue sarcomas, hepatotoxicity and other adverse events remain concerns. However, cases involving drug‐induced liver injury with ascites are rare.

**Case Presentation:**

We report a case of an 82‐year‐old woman with recurrent metastatic myxoid liposarcoma who developed simultaneous liver dysfunction and ascites after 12 cycles of trabectedin monotherapy. Laboratory tests revealed Grade 3 hepatic dysfunction, and imaging showed accumulation of ascitic fluid. In the absence of infection or thrombosis, a diagnosis of trabectedin‐induced liver injury with ascites was made. Supportive care, including hepatoprotective therapy, led to clinical improvement and resolution of the ascites.

**Discussion:**

Hepatotoxicity is a recognized adverse effect of trabectedin, with proposed mechanisms including direct hepatocellular injury and cytokine‐mediated damage. Peritonitis‐like symptoms may arise from ascites secondary to hepatic dysfunction. In this case, hepatic dysfunction due to trabectedin may be the trigger for ascites presenting as a peritonitis‐like manifestation.

**Conclusions:**

This case highlights the importance of vigilant monitoring during trabectedin therapy. Early recognition and management of liver injury and ascites presenting as a peritonitis‐like manifestation are crucial. Further research is warranted to elucidate the mechanisms and risk factors associated with these toxicities.

## 1 Introduction

Trabectedin is a synthetic antitumor agent derived from a natural compound originally isolated from the marine organism *Ecteinascidia turbinata* . The antitumor effects of trabectedin are mediated through its binding to the minor groove of DNA, leading to DNA damage, inhibition of transcriptional activity, and disruption of DNA repair mechanisms, particularly nucleotide excision repair, ultimately resulting in cell death [1]. Furthermore, trabectedin has been reported to selectively deplete tumor‐associated macrophages, thereby contributing to the improvement of the immunosuppressive tumor microenvironment [2]. Several clinical trials have demonstrated the efficacy of trabectedin, either as monotherapy or in combination with other agents, particularly, in patients with advanced soft tissue sarcomas and platinum‐sensitive recurrent ovarian cancer [3, 4]. More recently, promising efficacy has also been reported in patients with myxoid liposarcoma [3, 5]. The major adverse events associated with trabectedin include neutropenia, hepatic dysfunction (elevations of aspartate aminotransferase [AST] and alanine aminotransferase [ALT]), gastrointestinal symptoms (nausea and vomiting), and myalgia. These adverse events are generally manageable with supportive care, including the administration of granulocyte colony‐stimulating factor and hepatoprotective therapies [3, 4, 5, 6]. Liver injury typically presents with laboratory abnormalities such as elevations in transaminases and bilirubin, reflecting hepatocellular enzyme leakage and jaundice, whereas radiological abnormalities are extremely rare [3, 4, 6]. In particular, to the best of our knowledge, no previous case report has described the simultaneous development of laboratory abnormalities and ascites followed by a reduction and resolution of ascites in parallel with the improvement of liver injury. Here, we report a case in which these complications occurred concurrently but were successfully managed.

## 2 Case Presentation

An 82‐year‐old Japanese woman who had undergone surgical resection for myxoid liposarcoma of the right lower extremity at another hospital 5 years earlier was referred to our institution because of metastases to the lungs, liver, and pancreatic head in April 2024. Pathologically, the diagnosis of conventional myxoid liposarcoma with positivity for the FUS–DDIT3 fusion gene was established based on the surgical specimen from the primary tumor using an RT‐PCR method. The pathological findings of the peripancreatic metastatic lesions were also identical to those of the primary lesion (Supplementary Figure 1). She had received adjuvant chemotherapy with anthracycline and ifosfamide. Although multiple metastatic lesions were present, she had no apparent clinical symptoms or distress, including pain, fever, or organ dysfunction. The indolent biological behavior of the tumor may have accounted for the paucity of tumor‐related symptoms.

Neither lymphatic invasion of the intra‐abdominal lesions nor chylous ascites was identified at the time of biopsy of the pancreatic lesion. There was no history of hepatic or renal impairment. Despite prior postoperative adjuvant therapy with anthracycline and ifosfamide, both bone marrow and cardiac function were within normal ranges. As she was deemed a suitable candidate for salvage chemotherapy, trabectedin was administered at the standard dose of 1.2 mg/m^2^ every 3 weeks through a central venous port (CVP). Treatment was well tolerated and resulted in a clear reduction in tumor size. However, during the 12th cycle of trabectedin, she experienced unusually severe fatigue and loss of appetite, which she had not experienced previously. Subsequently, she developed right upper quadrant abdominal pain without fever. Blood tests revealed Grade 3 liver dysfunction (AST 207 IU/L, ALT 395 IU/L, D‐Bil 0.59 mg/dL, ALP 116 IU/L, γ‐GTP 444 IU/L), and contrast‐enhanced computed tomography (CT) revealed ascites (Figure 1A). There were no signs of inflammation around CVP, and no thrombus was detected at the catheter tip. Furthermore, CT revealed no alternative explanation for the elevated transaminase levels or ascites. Because these findings emerged on Day 7 of the chemotherapy cycle, a diagnosis of trabectedin‐induced liver injury and ascites presenting as a peritonitis‐like manifestation was made. Hepatoprotective therapy with ursodeoxycholic acid (300–600 mg/day) and Stronger Neo‐Minophagen C (60 mL/day) was initiated. Corticosteroids were not administered. On Day 10 of the chemotherapy cycle, chemotherapy‐induced pancytopenia progressed, leading to febrile neutropenia. Granulocyte colony‐stimulating factor (100 μg/day) and cefepime (4 g/day) were administered. Liver dysfunction and pancytopenia gradually improved over approximately 1 week (Figures 1B and 2), and by Day 15, the ascites had completely resolved (Figure 1C).

**FIGURE 1:**
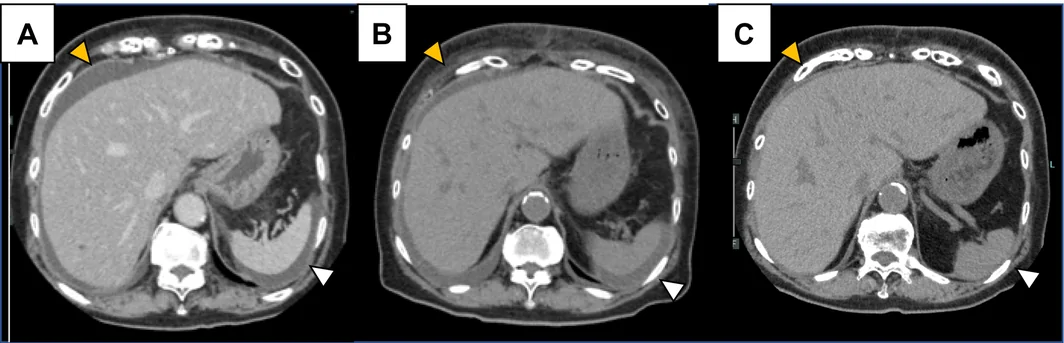
Abdominal CT revealed mild hepatomegaly and ascitic fluid (white arrowhead) accumulation around the liver and spleen were observed. (A) Abdominal CT revealed mild hepatomegaly and ascitic fluid accumulation around the liver and spleen. (B) By Day 5 after initiation of hepatoprotective therapy, the volume of ascites had decreased. (C) By Day 8 of treatment, the ascites had completely resolved.

**FIGURE 2:**
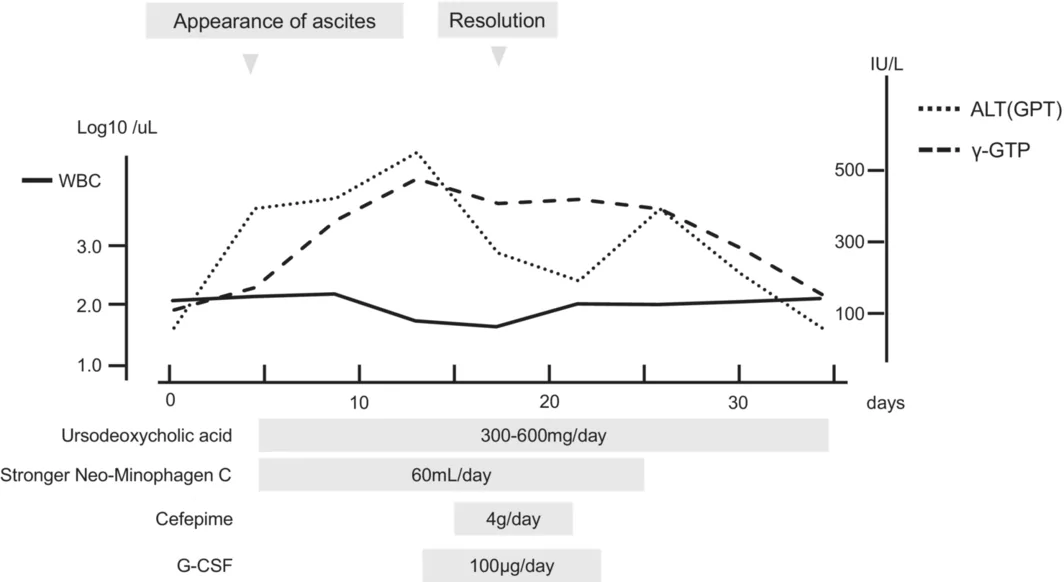
Clinical course of hepatic dysfunction and ascites, and the management of chemotherapy‐induced adverse events with supportive care. Ascites developed in association with elevated alanine aminotransferase (ALT) levels and resolved promptly following the normalization of ALT. Grade 3 leukopenia subsequently occurred, complicated by febrile neutropenia (FN), which was managed with granulocyte colony‐stimulating factor (G‐CSF) and cefepime. Although the patient's performance status temporarily declined to 2, it improved within 3 weeks in parallel with the resolution of FN and hepatic dysfunction. Days is the number of days after the 12th cycle of trabectedin administration.

Given this clinical course, further administration of trabectedin was deemed inappropriate, and the chemotherapy regimen was changed. As of December 2025, she had received three cycles of eribulin as an outpatient. Although she has mild anemia (hemoglobin 11.2 g/dL) and very mild peripheral neuropathy involving the bilateral soles, her activities of daily living were not impaired, and she remains fully independent. The most recent CT evaluation, performed in December 2025, showed continued tumor shrinkage, with a partial response maintained for 19 months from the initiation of trabectedin monotherapy.

## 3 Discussion

Trabectedin is an effective antitumor agent for soft tissue sarcomas, particularly those harboring chromosomal translocations [3].˒ [5] However, Grades 3–4 hepatic dysfunction occurs in approximately 10%–40% of patients, and rare cases of hepatic failure and treatment‐related death have been reported, highlighting the need for careful monitoring [1, 3, 4, 6]. In this context, the key finding in this case is the concurrent development of elevated liver enzymes and radiologically apparent ascites presentation that may mimic hepatic failure.

Trabectedin‐induced hepatotoxicity is thought to involve direct hepatocellular injury and activation of DNA damage‐response pathways. Dexamethasone premedication may attenuate these effects and reduce liver injury [7, 8, 9]. Hepatotoxicity usually presents as AST/ALT elevation, whereas trabectedin‐induced ascites presenting as a peritonitis‐like manifestation is rare, with a reported incidence of approximately 2.5%, which is less than one‐tenth the incidence of hepatotoxicity [10, 11]. To date, there are few reports describing the epidemiology or clinical course of trabectedin‐induced ascites and peritonitis, and the frequency of cases with concomitant liver injury and ascites remains unclear.

Several mechanisms have been proposed for trabectedin‐associated peritoneal inflammation, including catheter‐related microleakage, catheter‐associated thrombosis with drug reflux, and ascites secondary to drug‐induced hepatic dysfunction [9, 11, 12, 13]. In the present case, no evidence of catheter‐related inflammation, thrombosis, or drug leakage was observed. Given the concurrent onset and resolution of hepatic dysfunction and ascites (Figure 2), ascites formation secondary to liver impairment was considered the most plausible mechanism. Trabectedin‐induced liver injury is considered multifactorial, involving direct hepatocellular toxicity through DNA binding and transcriptional inhibition, metabolism‐related toxicity involving CYP3A4, and indirect effects on hepatic immune cells [9, 14]. The injury is usually reversible and manifests as transient elevations in transaminase levels. In cases of significant AST/ALT elevation, dose interruption or reduction is recommended, and dexamethasone premedication may also be effective [8, 9, 15].

In Japan, steroid premedication is not recommended for preventing trabectedin‐induced liver injury; therefore, corticosteroids were not administered in this case. Instead, Stronger Neo‐Minophagen C and ursodeoxycholic acid—both commonly used in Japan to treat chronic hepatitis C and drug‐ or heavy metal‐induced liver injury—were administered, and liver function subsequently improved [16, 17]. Collectively, these observations suggest that vigilance for hepatic injury is essential during trabectedin therapy and that early intervention may be crucial.

## 4 Conclusions

This report describes a single case, and further studies involving additional cases are needed. Nevertheless, this case highlights the possibility that trabectedin‐induced liver injury may be accompanied by ascites presenting with peritonitis‐like manifestations, although such concurrent presentations appear to be rare. Given that cases of hepatic failure have also been reported in clinical trials, early recognition of this potentially serious complication and prompt initiation of supportive care may be important in preventing progression to liver failure. Further case accumulation and investigation are warranted to identify prognostic factors and elucidate the underlying pathophysiological mechanisms in patients with liver injury accompanied by ascites, as well as to clarify the temporal relationship between trabectedin‐induced liver injury and the development of ascites.

## Author Contributions

**Tatsuyuki Kawahara:** conceptualization, investigation, formal analysis, writing – original draft. **Hiromichi Yamane:** conceptualization, investigation, formal analysis, writing – review and editing. **Ayaka Mimura:** investigation. **Hirohito Kirishi:** investigation. **Yusuke Sunada:** investigation. **Yoko Kosaka:** investigation. **Naruhiko Ichiyama:** investigation. **Yasunari Nagasaki:** investigation. **Nobuaki Ochi:** formal analysis. **Hidekazu Nakanishi:** investigation. **Hideyo Fujiwara:** investigation. **Nagio Takigawa:** supervision, writing – review and editing.

## Funding

This work was supported in part by JSPS KAKENHI (Grant JP22K09436).

## Ethics Statement

This study was approved by the Ethics Committee of the Kawasaki Medical School (No 6284‐00) and was conducted in accordance with the Declaration of Helsinki principles.

## Consent

The patient provided informed consent for publication with written informed consent format.

## Conflicts of Interest

Although all authors declare that no conflicts of interest exist, including any financial and personal relationships with other people or organizations that could inappropriately influence our work, Department of General Internal Medicine 4 in Kawasaki Medical School has received the research support grants from Takeda Pharmaceutical Co Ltd., Boehringer Ingelheim Japan Co Ltd., Taiho Pharmaceutical, Chugai Pharmaceutical Co Ltd., Kyowa Hakko Kirin Co Ltd., Pfizer Japan Inc., Eli Lilly Japan K.K, Daiichi Sankyo Co Ltd., Ono Pharmaceutical Co Ltd., and Nippon Kayaku Co Ltd.

## Supporting information


**Supplemental Figure 1.** Both the primary tumor and the metastatic lesion (peripancreatic) demonstrated morphological features consistent with conventional myxoid liposarcoma. Immunohistochemical staining revealed that both CDK4 and MDM2 were negative.

## Data Availability

The data that support the findings of this study are available on request from the corresponding author. The data are not publicly available due to privacy or ethical restrictions.
