# Supplementary material for: Ascites Presenting as a Peritonitis‐Like Manifestation and Liver Injury due to Trabectedin Monotherapy in a Patient With Myxoid Liposarcoma
Source: Cancer Rep (Hoboken). 2026 Oct 31;9(11):e70740. doi: 10.1002/cnr2.70740 (PMC13722948; doi:10.1002/cnr2.70740)
Supplement: Supplementary file 1 [file CNR2-9-e70740-s001.docx]

Supplementary Figure 1

H-E (**x400**)

Immunohistochemistry

**（CDK4　X400）**

Immunohistochemistry

**(MDM2	x400)**

H-E (**Roupe**)
